# Repurposed Analog of GLP-1 Ameliorates Hyperglycemia in Type 1 Diabetic Mice Through Pancreatic Cell Reprogramming

**DOI:** 10.3389/fendo.2020.00258

**Published:** 2020-05-13

**Authors:** Adrian Villalba, Silvia Rodriguez-Fernandez, David Perna-Barrull, Rosa-Maria Ampudia, Laia Gomez-Muñoz, Irma Pujol-Autonell, Eva Aguilera, Mireia Coma, Mary Cano-Sarabia, Federico Vázquez, Joan Verdaguer, Marta Vives-Pi

**Affiliations:** ^1^Immunology Section, Germans Trias i Pujol Research Institute, Autonomous University of Barcelona, Badalona, Spain; ^2^Endocrinology Section, Germans Trias i Pujol Research Institute, Autonomous University of Barcelona, Badalona, Spain; ^3^Anaxomics Biotech SL, Barcelona, Spain; ^4^Catalan Institute of Nanoscience and Nanotechnology, CSIC and The Barcelona Institute of Science and Technology, Bellaterra, Spain; ^5^Immunology Unit, Department of Experimental Medicine, Faculty of Medicine, IRBLleida, University of Lleida, Lleida, Spain; ^6^CIBER of Diabetes and Associated Metabolic Disease (CIBERDEM), ISCIII, Madrid, Spain

**Keywords:** beta cell regeneration, neogenesis, transdifferentiation, liraglutide, drug repositioning

## Abstract

Type 1 diabetes is an autoimmune disease caused by the destruction of the insulin-producing β-cells. An ideal immunotherapy should combine the blockade of the autoimmune response with the recovery of functional target cell mass. With the aim to develop new therapies for type 1 diabetes that could contribute to β-cell mass restoration, a drug repositioning analysis based on systems biology was performed to identify the β-cell regenerative potential of commercially available compounds. Drug repositioning is a strategy used for identifying new uses for approved drugs that are outside the scope of the medical indication. A list of 28 non-synonymous repurposed drug candidates was obtained, and 16 were selected as diabetes mellitus type 1 treatment candidates regarding pancreatic β-cell regeneration. Drugs with poor safety profile were further filtered out. Lastly, we selected liraglutide for its predictive efficacy values for neogenesis, transdifferentiation of α-cells, and/or replication of pre-existing β-cells. Liraglutide is an analog of glucagon-like peptide-1, a drug used in patients with type 2 diabetes. Liraglutide was tested in immunodeficient NOD-*Scid IL2rg*^−/−^ (NSG) mice with type 1 diabetes. Liraglutide significantly improved the blood glucose levels in diabetic NSG mice. During the treatment, a significant increase in β-cell mass was observed due to a boost in β-cell number. Both parameters were reduced after withdrawal. Interestingly, islet bihormonal glucagon^+^insulin^+^ cells and insulin^+^ ductal cells arose during treatment. *In vitro* experiments showed an increase of insulin and glucagon gene expression in islets cultured with liraglutide in normoglycemia conditions. These results point to β-cell replacement, including transdifferentiation and neogenesis, as aiding factors and support the role of liraglutide in β-cell mass restoration in type 1 diabetes. Understanding the mechanism of action of this drug could have potential clinical relevance in this autoimmune disease.

## Introduction

An essential requirement to cure type 1 diabetes is the recovery of β-cells lost after the autoimmune destruction. The pancreas can restore β-cells from different sources, after injury (1) or drug administration (2, 3) in mice, and upon pathophysiological conditions in humans (4). With the aim to develop new therapies for β-cell replacement, a repositioning analysis based on systems biology was performed to identify the regenerative potential of commercially available compounds.

Drug repositioning is a strategy for identifying new uses for approved drugs that are outside the scope of the medical indication. This approach uses a bioinformatics tool, based on networks of drugs, proteins, and diseases, which screens approved compounds that can be repurposed for other diseases (5). This has resulted in successful drug discovery for diseases (6) such as cancer (7) and Alzheimer's disease (8). Given that, we aimed to look for drugs that can induce β-cell replacement through α-cell to β-cell transdifferentiation (9), neogenesis from multipotent ductal progenitors (1), and/or replication of pre-existing β-cells (10). This resulted in the identification of liraglutide—an analog of glucagon-like peptide-1 (aGLP-1), a drug used in patients with type 2 diabetes (11), especially those with obesity (12).

GLP-1 is produced in the gut after food intake and acts by increasing insulin release. Liraglutide ameliorates insulin resistance in type 2 diabetes models (13, 14). Recently, GLP-1 has been shown to promote transdifferentiation from α-cells to β-cells (15, 16).

We report here that liraglutide ameliorates hyperglycemia in mice with type 1 diabetes by inducing insulin^+^glucagon^+^ cells and insulin-producing cells from the pancreatic ducts. This is the first description of an approved drug—identified by a repositioning—that promotes the generation of insulin-expressing cells from pancreatic ducts and ameliorates hyperglycemia in experimental type 1 diabetes.

## Materials and Methods

### Systems Biology Analysis for Drug Discovery and Repositioning Analysis

The therapeutic performance mapping system is a top-down systems biology approach based on artificial intelligence and pattern recognition that integrates all available pharmacological knowledge to create mathematical models that simulate human pathophysiology *in silico*. The methodology employed has been previously described (17) and applied elsewhere (18, 19).

A manually curated list of proteins known to be involved in the mechanisms of β-cell regeneration—α-cell to β-cell transdifferentiation, neogenesis from ductal precursors, and β-cell replication—was obtained (Supplementary Table 1) and used for focusing the analysis toward β-cell regeneration in a human biological network context.

The human biological network created incorporated the available relationships (edges or links) between proteins (nodes) from a regularly updated in-house database drawn from public sources: KEGG (20, 21), REACTOME (22), INTACT (23), BIOGRID (24), HPRD (25), and TRRUST (26). All information of the key proteins defined during the molecular and the biological characterization and stored in relevant databases (drug targets, other diseases effectors, biomarkers…) was incorporated into the biological networks (27).

Artificial neural networks (ANNs) are supervised algorithms that identify relations between proteins (e.g., drug targets) and clinical elements of the network (18, 19) by inferring the probability of the existence of a specific relationship between two or more protein sets. This is based on a validation of the predictive capacity of the model toward the truth table, a selected collection of known input (drug targets)–output (indications) relationships defined through specific scientific literature search and hand-curated assignment of proteins to the conditions included in the biological effector database (17). The learning methodology used consisted of an architecture of stratified ensembles of neural networks as a model, trained with a gradient descent algorithm to approximate the values of the given truth table. The neural network model used was a multilayer perceptron (MLP) neural network classifier. The MLP gradient descent training depends on randomization initialization and, to avoid random errors, 1,000 MLPs are trained with the training subset and the best 100 MLPs are used. In order to correctly predict the effect of a drug independently of the number of targets, different ensembles of neural networks are trained for different subsets of drugs according to their number of targets (drugs with one target, two targets, three targets…). Then, the predictions for a query drug are calculated by all the ensembles and pondered according to the number of targets of the query drug (the difference between the number of targets of the query and the number of targets of the drugs used to calculate each ensemble is used to ponder the result of each ensemble). A cross-validation with the truth table information showed that the accuracy of the described ANNs to reproduce the indications compiled in DrugBank (28, 29) is 81.23% for those drugs with all targets in the human biological network. ANNs were used to screen the predicted relationship of 6,605 different drugs toward the β-cell regeneration molecular definition. The repurposed drug candidates were sorted and selected by its relationship with β-cell regeneration mechanisms.

### Mice and Diabetes Induction

NOD mice, immunodeficient NOD.SCID-IL2Rγ^−/−^ (NSG) mice, and C3HeB/FeJ mice were bred in our own facility. The NOD and NSG mice were kept under specific pathogen-free conditions. The NSG model was selected for its lack of an adaptive immune system, resulting in the absence of autoimmunity, and allowing to determine the raw effect of the drug. Type 1 diabetes was induced in NSG mice at 8–14 weeks of age by a single i.p. injection of streptozotocin (STZ, 150 mg/kg) (Sigma-Aldrich). Type 1 diabetes was confirmed at 48–72 h post-STZ injection, after either two successive 2-h fasting blood glucose levels higher than 250 mg/dl or with one higher than 300 mg/dl. The mice were euthanized by cervical dislocation. All animal studies were approved by the institutional animal ethics committee.

### Treatment With aGLP-1

Immediately after type 1 diabetes diagnosis, the NSG mice (*n* = 12 mice/group) were treated with liraglutide (Victoza®, Novo Nordisk A/S), injected (s.c.) daily up to 30 days, following the dosage of 0.3 mg/kg at day 1, 0.6 mg/kg at day 2, and 1 mg/kg from day 3 onwards as described (30). After the withdrawal of the liraglutide treatment, the mice were maintained for 5 days. The control group (*n* = 6 mice) received phosphate-buffered saline (PBS). Blood glucose was determined twice weekly, after 2 h of fasting, throughout the study.

### Intraperitoneal Glucose Tolerance Test and Insulin Tolerance Test

Intraperitoneal glucose tolerance test (IPGTT) was performed in fasting conditions in the three groups: (1) diabetic NSG mice responding to liraglutide after 15 days of treatment (Lira, *n* = 3), (2) untreated diabetic and hyperglycemic NSG mice (T1D, *n* = 3), and (3) healthy and normoglycemic NSG mice (sham, *n* = 3). At point 0, basal glucose level was determined. The mice were subsequently given an i.p. injection of 2 mg of glucose (Sigma-Aldrich) per gram of body weight and glycemia was measured after 15, 30, 60, 120, and 210 min. Insulin tolerance test (ITT) was performed in fasting conditions in 8-week-old and normoglycemic NOD mice and C3HeB/FeJ mice injected s.c. with insulin (0.5 U/kg, *n* = 3) or liraglutide (1 mg/kg, *n* = 3). Glycemia was determined after 15, 30, and 60 min.

### Immunofluorescence Staining and Histometric Analysis

Immunofluorescence staining was performed to identify pancreatic insulin-producing cells in a minimum of three mice per condition. Briefly, the pancreas were harvested and snap-frozen in an isopentane/cold acetone bath. A minimum of eight cryostat sections (5 μm) from every organ were sequentially stained by indirect immunofluorescence with antibodies to insulin, glucagon, CK19 (Sigma-Aldrich), or Pdx1 (Abcam) and FITC- or TRITC-labeled secondary antibodies (Sigma-Aldrich) as described (31). The nuclei were stained with Hoechst (Invitrogen). The samples were observed in a fluorescence microscope and analyzed (ImageJ Software) (32). For histometric analysis, six mice per group were used. To determine the β-cell counts, one section every 150 μm of tissue was sampled as described (33), resulting in 12–16 sections per pancreas. The β-cell mass was calculated by multiplying the relative insulin^+^ area per total pancreas weight, and the β-cell number as well as the insulin^+^ aggregates were calculated by manually counting the nuclei within the insulin^+^ area and extrapolating to the whole organ as previously described (34). The β-cell size was assessed by dividing the insulin^+^ area per total nuclei (34). The intensity of fluorescence was measured in arbitrary units using Fiji (32).

To determine the insulin^+^glucagon^+^ cells, pancreas from three mice from each group were analyzed (T1D, Lira 48 h, Lira, post-Lira, and sham). Briefly, 12 non-overlapping pancreatic cryostat sections from each mouse were stained for insulin and glucagon. A minimum of 72 islets per mouse was considered and the percentage of islets that contained bihormonal cells was determined. To assess ductal insulin^+^ cells, pancreas from four mice from each group were analyzed (T1D, sham, and Lira). Briefly, four non-overlapping pancreatic cryostat sections from each mouse were stained for CK19 and insulin. A minimum of 23 ductal areas *per section* was considered and the percentage of ducts that contained insulin^+^ cells was determined. To prove the colocalization of insulin and glucagon in islet cells and insulin and CK19 in ductal cells, confocal microscopy was performed using an Axiobserver Z1 (Zeiss) and by analyzing 1-μm sections.

### *In vivo* Tracking of Liraglutide

Liraglutide was conjugated to AlexaFluor750 (AF750, Invitrogen) using a standard method (Thiol-Reactive Probes, Invitrogen). *In vivo* and *ex vivo* near-infrared fluorescence imaging was performed (Pearl Impulse imaging system, LI-COR) in NOD mice anesthetized with ketamine (50 mg/kg) and xylazine (5 mg/kg) at 15 and 60 min after the s.c. administration of 1 mg/kg of AF750-liraglutide in 50 μl of PBS. At the end of each checkpoint, spleen, stomach, fat, heart, liver, pancreas, lungs, kidney, salivary glands, thymus, and bladder were imaged *ex vivo*. Fluorescent signal intensity was semi-quantitatively assessed: the levels were normalized by subtracting the background and represented as a relative index of fluorescence in each organ per gram of tissue.

### Islets and β-Cell Line

Islets from young non-diabetic NOD mice (*n* = 7) were obtained from pancreas digested with collagenaseP (2.5 mg/ml in PBS; Sigma-Aldrich), after having been injected through the common bile duct, resected, and incubated at 37°C for 30 min. The pancreas was dissociated and the islets were hand-picked. Groups of 50 islets were cultured in a medium of high (10 mM, RPMI-1640, Biowest) or normal glucose concentration (6.1 mM, Ham F-10, Gibco) as described (35), both in basal conditions—culture media—and by adding liraglutide at 1,000 nM for 48 h.

The β-cell line NIT-1 (ATCC) (36) was cultured (35) with liraglutide at 10, 100, and 1,000 nM for 12, 24, and 48 h. Viability was assessed with annexin V (AnnV) PE (Immunotools) and 7-amino-actinomycin D (7aad) labeling (BD Pharmingen) and analyzed with FACS Canto II (BD Biosciences).

### Gene Expression Analysis

To determine the effects of liraglutide on gene expression, quantitative RT-PCR was performed. Briefly, RNA was isolated from islets using RNeasy Micro Kit (Qiagen) and reverse-transcribed with a cDNA Reverse-Transcription Kit (ThermoFisher Scientific). cDNA synthesis was performed using random hexamers (0.5 mg/ml, BioTools) and reverse transcriptase Moloney–murine–leukemia–virus (200 U/ml, Promega). Targeted cDNA was pre-amplified with TaqMan PreAmp MasterMix (ThermoFisher Scientific). Quantitative RT-PCR assays were performed with TaqMan universal assay (ThermoFisher Scientific) on a LightCycler®480 (Roche) using the following TaqMan assays: *Ins2* (Mm00731595_gH), *Gcg* (Mm00801714_m1), *Ki67* (Mm01278617_m1), and *Il17a* (Mm00439618_m1). The expression for each gene of interest was normalized to that of the housekeeping gene *Gapdh* (Mm99999915_g1), as described in the 2^−Δ*Ct*^ method (37). Values from islets were normalized using their respective basal controls and represented as a ratio (relative gene expression).

### Effect of Liraglutide on NIT-1 Cell Line

NIT-1 cells were stained with anti-CD44 BV786 (BD Biosciences), anti-class I major histocompatibility complex (MHC) eFluor-450 (eBioscience), anti-CD14 PE, and anti-CD49b FITC (Immunotools). Viability was assessed with AnnV PE (Immunotools) and 7aad (BD Biosciences) as detailed above. Median fluorescence intensity and viability were determined using flow cytometry (LSR Fortessa, BD Biosciences). Corresponding fluorescence minus one staining was used as control. The data were analyzed using FlowJo (Tree Star Inc).

### Statistical Analysis

Prism 7.0 (GraphPad Software Inc.) was used to perform the statistical analyses. For comparisons of unpaired data, a non-parametric Mann–Whitney test was used. The statistical tests applied to each data set are specified in each figure legend.

## Results

### Liraglutide Is a Repurposed Candidate for β-Cell Regeneration

A total of 28 non-synonymous repurposed drug candidates showed a *p* < 0.05 (predicted value ≥76.075%), and 16 were selected as type 1 diabetes treatment candidates in the context of pancreatic β-cell regeneration. Drugs with poor safety profile were further filtered out. Lastly, we selected liraglutide (predicted value of 96.88%) that fulfilled all the criteria and had a novel, potentially beneficial mechanism toward β-cell regeneration.

### Liraglutide Improves Hyperglycemia in Diabetic Mice

**Figure 1:**
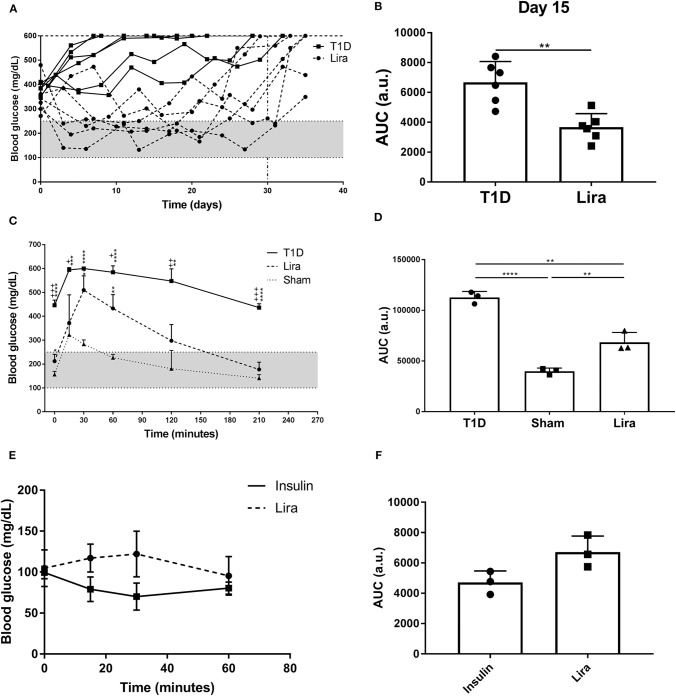
Effect of liraglutide in diabetic immunodeficient NOD.SCID-IL2Rγ^−/−^ (NSG) mice. **(A)** Two-hour fasting blood glucose levels (mg/dl) in mice rendered diabetic by a single dose of streptozotocin (150 mg/kg) and then treated with daily s.c. injections of liraglutide (Lira responders, circles) and phosphate-buffered saline (T1D, squares) from day 0 to 30 (dashed line). The filled area corresponds to normoglycemia levels in mice. **(B)** Area under the curve (AUC) of the graph in **(A)** at day 15, when all animals remain alive. Results are mean ± SD, and differences were found between groups (**p < 0.01, Mann–Whitney test). **(C)** Intraperitoneal glucose tolerance test in diabetic NSG mice treated with daily s.c. liraglutide for 15 days (Lira, dashed line), non-treated diabetic animals (T1D, continuous line), and normoglycemic mice (sham, dotted line). Statistical differences were found between Lira and T1D groups (+*p* < 0.05, ++*p* < 0.01, + + +*p* < 0.001, Mann–Whitney test) and in both groups when compared to sham (dotted line) (*p < 0.05, **p < 0.01, ***p < 0.001, ****p < 0.0001, Mann–Whitney test). The filled area corresponds to normoglycemia levels in mice. **(D)** AUC of the graph in **(C)**. The results are mean ± SD, and differences were found between groups (**p < 0.01, ****p < 0.0001, Mann–Whitney test). **(E)** Insulin tolerance test performed in normoglycemic mice injected with Lira (1 mg/kg, dashed line) or insulin (0.5 U/kg, continuous line). **(F)** AUC of the graph in **(E)**. The results are mean ± SD; no statistical differences were found between groups.

We determined the effect of liraglutide in immunodeficient NSG mice because of their absence of autoimmunity and low phenotypic heterogeneity. Type 1 diabetes was induced in NSG mice by a single STZ injection. All mice showed disease symptoms at 48–72 h after administration. A total of 50% (six of 12) of the treated NSG mice were responders to liraglutide according to the improvement of blood glucose levels during treatment. The remaining six mice did not have decreased blood glucose levels in any way. No differences in terms of initial fasting glycemia (mg/dl) were found between the responder (346.8 ± 72.5, mean ± SD) and the non-responder (397.7 ± 57.71) mice. The NSG mice with diabetes partially recovered normoglycemia during liraglutide administration until day 30 (Lira responders) when compared to the non-treated mice (T1D) (Figure 1A). After treatment withdrawal, all mice became hyperglycemic. A statistical difference between the treated and the control groups was observed when analyzing the area under the curve (AUC) after 15 days of treatment (Figure 1B). The response of the NSG mice to liraglutide after an acute increase in blood glucose levels showed that the treated diabetic mice (Lira) recovered normoglycemia at 210 min after glucose injection similarly to the non-diabetic group, whereas the diabetic non-treated mice remained hyperglycemic (>400 mg/dl, T1D) (Figure 1C). Significant differences found between the treated and the non-treated mice demonstrate that insulin production and secretion improve by the effect of this aGLP-1. The analysis of AUC showed an intermediate response to glucose stimulation in the liraglutide-treated group (Figure 1D). To elucidate whether liraglutide acts in a similar manner as insulin administration, an ITT was performed in normoglycemic NOD mice. The group treated with insulin displayed a reduction in glycemia after 30 min that was maintained until 60 min. By contrast, the liraglutide-treated group displayed an increase in glycemia until 30 min, which was normalized at 60 min similarly to the insulin-treated animals (Figure 1E). Despite that no significant differences were observed, the opposite tendency between both groups observed in the AUC (Figure 1F) revealed that liraglutide and insulin do not act similarly. To determine the effect of the genetic background, the acute effect of liraglutide was also determined on the C3HeB/FeJ strain. The results showed an insulinotropic effect of liraglutide in this strain of mice (Supplementary Figure 1) with a tendency to differ at the endpoint (60 min) in comparison to insulin.

### Liraglutide Transiently Increases the β-Cell Mass

**Figure 2:**
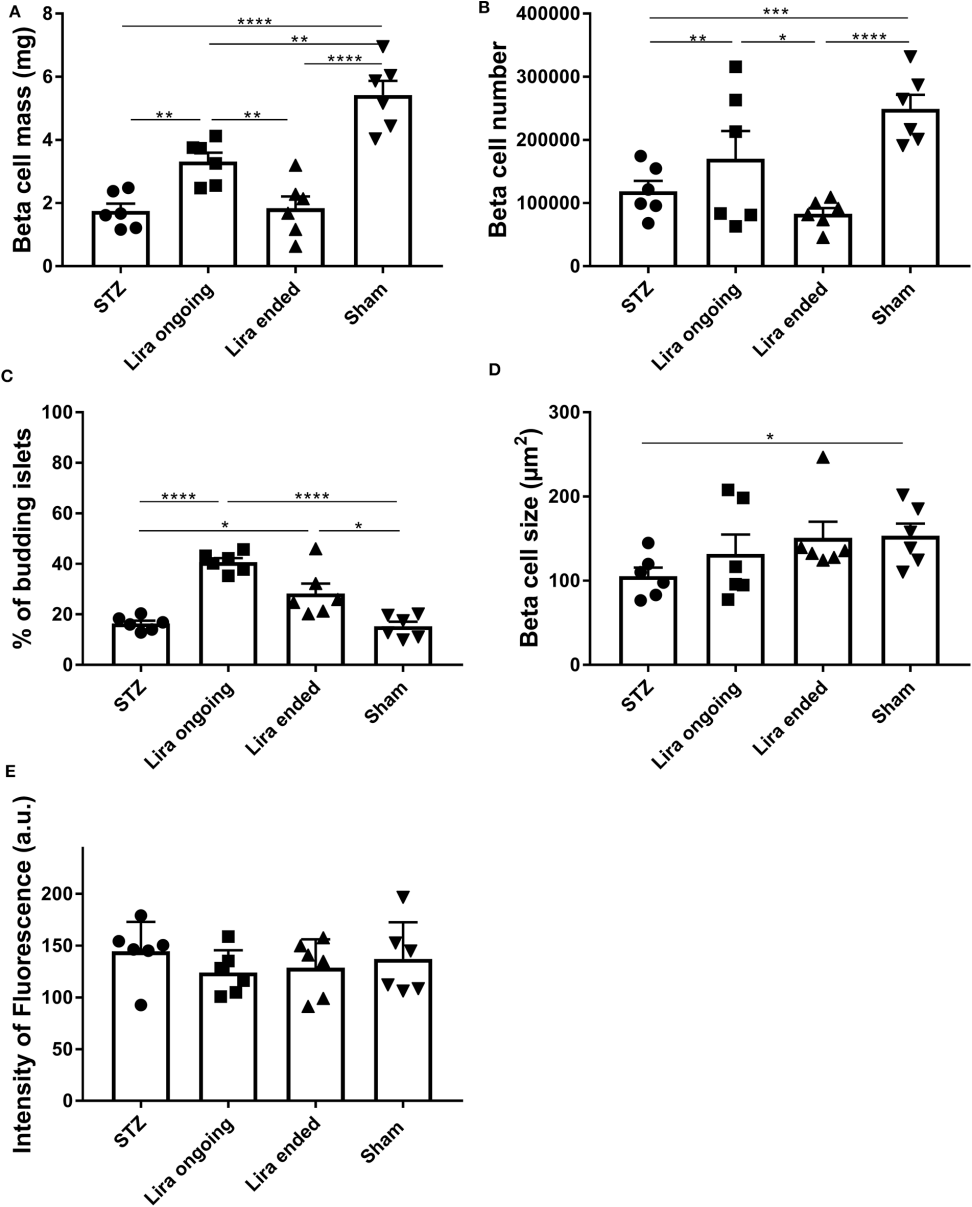
Effect of liraglutide in the endocrine pancreas of diabetic immunodeficient NOD.SCID-IL2Rγ^−/−^ (NSG) mice. **(A)** β-cell mass (mg), **(B)** β-cell number, **(C)** percentage of budding islets (arising from ducts), and **(D)** β-cell size (μm^2^) from NSG mice rendered diabetic by streptozotocin and treated with phosphate-buffered saline (T1D), with daily s.c. injections of liraglutide during 7–15 days (Lira), 5 days after the withdrawal of the liraglutide treatment (post-Lira) and normoglycemic mice (sham). The results are mean ± SD, and differences were found between groups (*p < 0.05, **p < 0.01, ***p < 0.001, ****p < 0.0001, Mann–Whitney test). **(E)** Intensity of fluorescence of insulin staining in 5-μm cryostat pancreatic sections of NSG mice. The intensity was measured for all the islets analyzed per animal, and the mean of the intensities of all the islets is represented as a symbol. The results are mean ± SD, and no statistical differences were found between groups (Mann–Whitney test).

The β-cell mass of the NSG mice after 7–15 days of treatment with liraglutide significantly increased when compared to that of the diabetic non-treated mice, although they did not reach normal levels (Figure 2A). This effect was lost 5 days after withdrawal. In this sense, an increase in β-cell number was detected during the treatment in comparison to T1D and post-treatment groups (Figure 2B). These alterations were maintained even with normalized values to body weight or pancreatic tissue (Supplementary Table 2). Similarly, the percentage of islets emerging from ducts was increased both during treatment and after therapy removal (Figure 2C). These alterations were not related to changes in either β-cell size (Figure 2D) or insulin fluorescence intensity (33) (Figure 2E).

### Liraglutide Induces Endocrine Cell Rearrangement in the Pancreas

**Figure 3:**
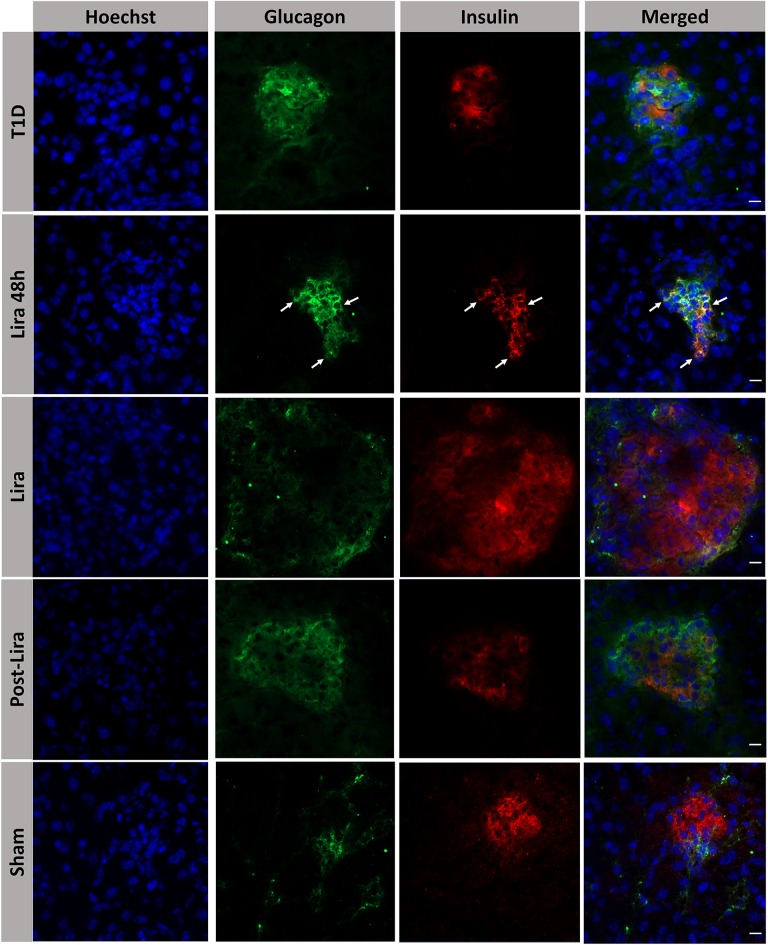
Assessment of the effect of liraglutide in the induction of bihormonal insulin^+^ glucagon^+^ cells. Triple immunofluorescence staining of 5-μm cryostat pancreatic sections for α-cells (glucagon, green) and β-cells (insulin, red) in immunodeficient NOD.SCID-IL2Rγ^−/−^ mice rendered diabetic by streptozotocin and treated with phosphate-buffered saline (T1D), or with liraglutide for 48 h (Lira 48 h), for 7–15 days (Lira) and after withdrawal of liraglutide at day 30 (post-Lira). Normoglycemic mice were included as control (sham). The white arrows in Lira 48 h staining depict bihormonal cells. The nuclei in all pictures were stained with Hoechst (blue). The scale bar indicates 5 μm.

The histological analysis revealed the presence of bihormonal cells (insulin^+^glucagon^+^) in the islets of NSG mice at the beginning of the treatment with liraglutide but not after 7–15 days or withdrawal (Figure 3). A total of 48.14 ± 1.53% of islets contained bihormonal cells, and the percentage of insulin^+^glucagon^+^ cells in relation to the total number of beta cells in the islets was 11.38 ± 2.97%. No bihormonal cells were detected in the other groups. Confocal microscopy images prove the colocalization of insulin and glucagon in the same cell (bihormonal cells) (Supplementary Figure 2).

**Figure 4:**
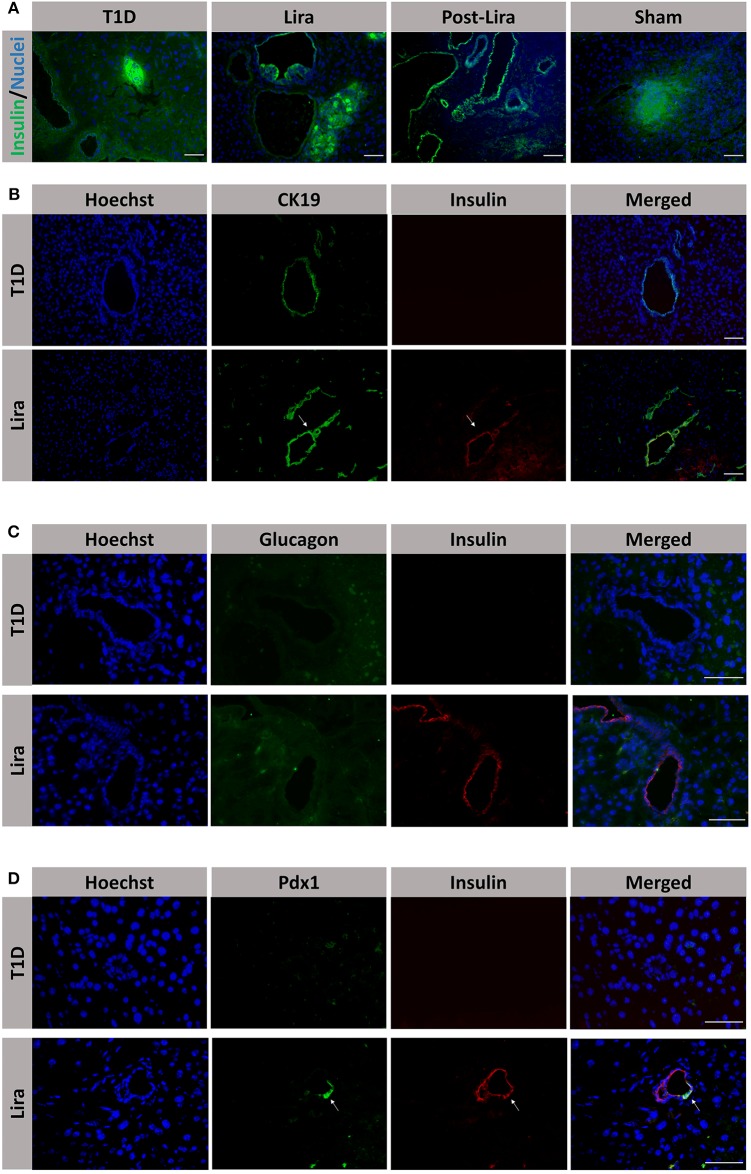
Assessment of the effect of liraglutide in the induction of insulin-expressing ducts. **(A)** Staining for β-cells in the pancreas of immunodeficient NOD.SCID-IL2Rγ^−/−^ (NSG) mice rendered diabetic by streptozotocin and treated with phosphate-buffered saline (PBS; T1D), or with liraglutide for 48 h (Lira 48 h), for 7–15 days (Lira) and after the withdrawal of liraglutide at day 30 (post-Lira). The Lira and post-Lira groups show the presence of neo-islets emerging from the ducts. Normoglycemic mice were included as control (sham). **(B)** Staining for ductal cells (CK19, green) and β-cells (insulin, red) in the same groups than in **(A)**. The white arrow depicts a CK19^+^ duct that is positive for insulin expression. **(C)** Staining for glucagon (green) and insulin (red) in ductal cells of animals treated with liraglutide for 7–15 days (Lira). **(D)** Staining for Pdx1 (green) and insulin (red) in the ductal part of diabetic NSG mice treated with PBS (T1D) or liraglutide for 7–15 days (Lira). The white arrows depict positivity for both Pdx1 and insulin in the ducts. The nuclei in all pictures are stained with Hoechst (blue). The scale bar in all pictures indicates 50 μm.

Moreover, the pancreatic sections of the NSG mice treated for 7–15 days and after withdrawal revealed the existence of insulin^+^ bodies emerging from ducts and without resembling the classical islet shape (Figure 4A). Between 23 and 47 ducts (CK19+) were analyzed in every pancreatic section. The percentage of ducts that contained insulin^+^ cells was 50.83 ± 7.31% in mice treated with liraglutide (Lira group), whereas no ducts with insulin^+^ cells were detected in normoglycemic mice (sham group, 0%) or untreated T1D mice (T1D, 0%). The ducts that contained insulin^+^ cells were analyzed and, in these ductal areas, 82.85 ± 4.37% of doble-positive CK19^+^ insulin^+^ cells were found. Interestingly, the ductal areas positive for insulin in mice treated with liraglutide were detected from 48 h to the end of the treatment and even 5 days after the withdrawal (Figure 4B, white arrows). Confocal microscopy images prove the colocalization of insulin and CK19 in the same cell (Supplementary Figure 3). These insulin^+^CK19^+^ cells were glucagon-negative (Figure 4C). Moreover, we assessed the expression of the β-cell marker Pdx1 in ductal cells, identifying a subpopulation of Pdx1^+^ cells in pancreatic ducts from treated animals (white arrow, Figure 4D).

**Figure 5:**
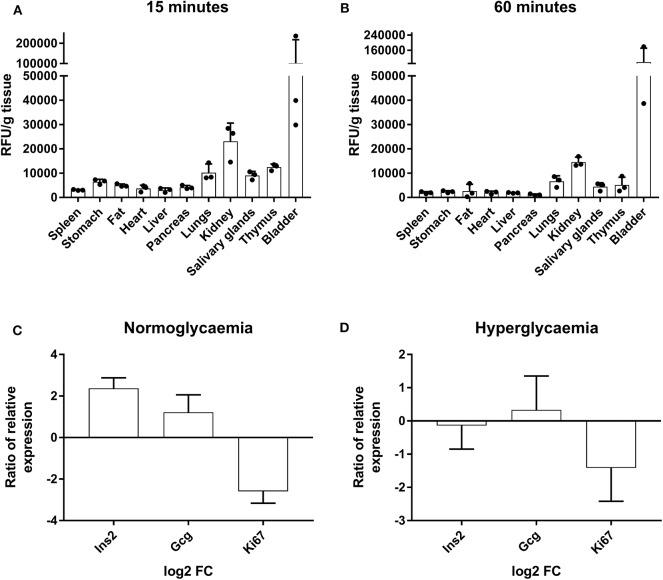
Tracking of AF750-labeled liraglutide in prediabetic NOD mice and determination of gene expression in islets of Langerhans. **(A)** Histogram of *ex vivo* fluorescent signal relative to the grams of tissue of several organs from NOD mice at 15 min and **(B)** at 60 min after a s.c. injection of AF750-labeled liraglutide. The results are mean ± SEM of three independent experiments. **(C,D)** Ratio of relative gene expression of the selected genes in isolated islets of Langerhans after 48 h of 1,000-nM liraglutide treatment (*n* ≥ 3) in relation to basal conditions analyzed by qRT-PCR in conditions of normoglycemia and hyperglycemia. Gene expression was normalized to *Gapdh*. The bars show the mean ± SD of the log2 of fold change using basal transcription as standard value.

To determine if liraglutide effects could be due to the accumulation in the pancreas, its biodistribution was assessed. The *in vivo* tracking of AF750-liraglutide showed an affinity for several organs including the pancreas that was higher at 15 min (Figure 5A) than at 60 min (Figure 5B), revealing an acute effect.

To further investigate the short-term effects of liraglutide, the expression of specific genes was assessed in islets from healthy NOD mice (three to four per group) cultured with or without liraglutide for 48 h in normal and high glucose concentrations. Previously, and in order to discard autoreactive insulitis in the islets, the expression of *Il17a* was assessed and found negative (data not shown). After exposure to liraglutide, a trend to upregulate *Gcg* and to downregulate *Ki67* was observed both at doses of 6.1 mM glucose (Figure 5C) and 10 mM glucose (Figure 5D). Finally, after exposure to liraglutide, the insulin gene (*Ins2*) appeared upregulated in normal glucose concentration conditions and slightly downregulated in high glucose concentration conditions.

### Liraglutide Alters Membrane Molecule Expression in NIT-1 Cell Line

**Figure 6:**
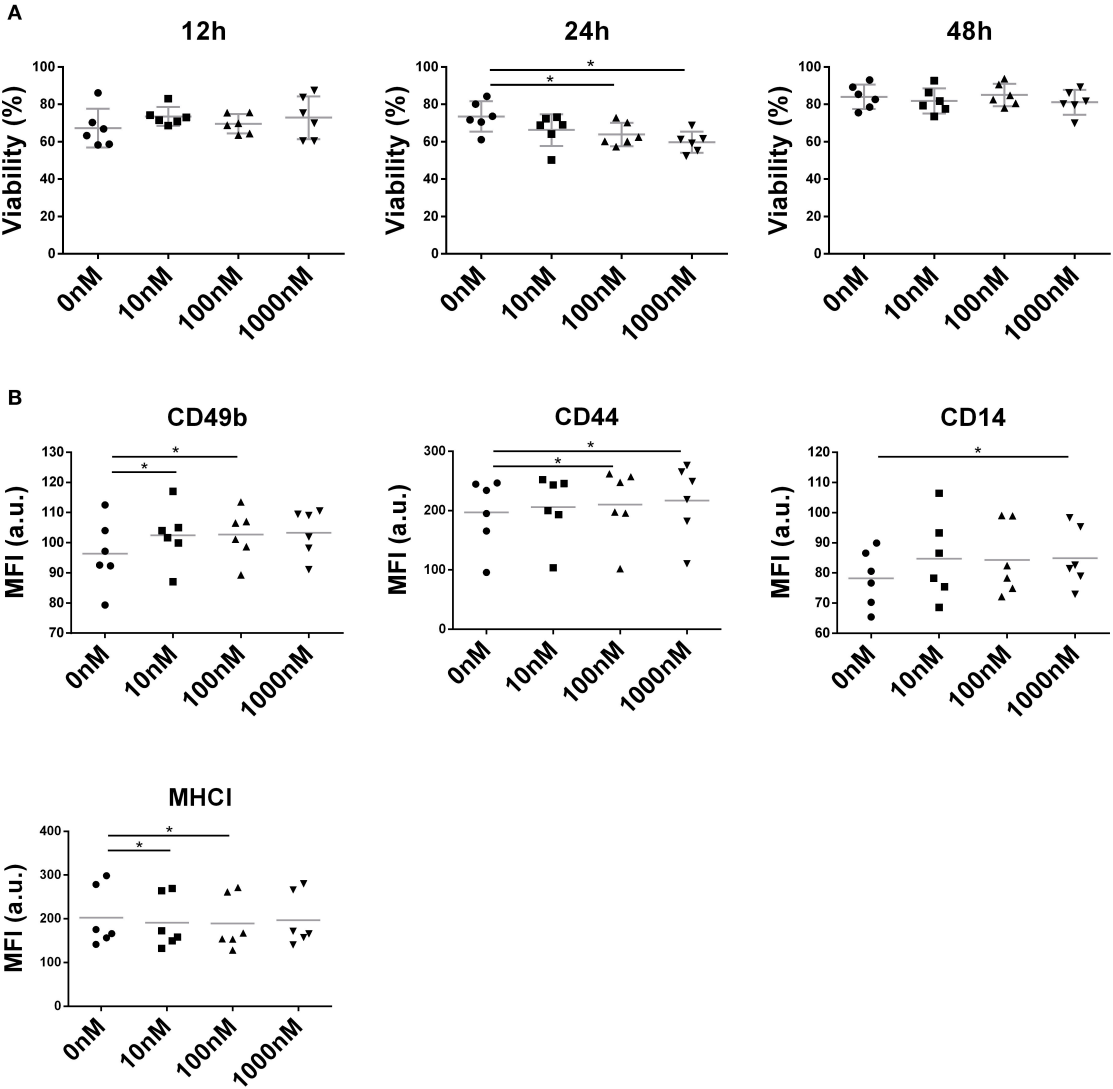
Effect of liraglutide in the NIT-1 β-cell line. **(A)** Percentage of viable NIT-1 cells (annexin V PE^−^, 7aad^−^) after 12, 24, and 48 h of co-culture with increasing liraglutide concentrations (10, 100, and 1,000 nM) and control condition (*p < 0.05, Mann–Whitney test). **(B)** Median of fluorescence intensity for CD49b, CD44, CD14, and MHC class I surface expression (*p < 0.05, Mann–Whitney test).

NIT-1 cell viability remained unaffected by liraglutide (10, 100, and 1,000 nM) at both 12 and 48 h (Figure 6A) but showed a deleterious effect on the NIT-1 cell line at 100 and 1,000 nM during 24 h (Figure 6A). Interestingly, liraglutide increased the expression of adhesion molecules such as CD49b, CD44 (Figure 6B), and CD14, a receptor of the innate immunity, and reduced the expression of MHC class I.

## Discussion

Drug repositioning is an attractive strategy for identifying new uses for approved drugs. The algorithm of this method was fed with target proteins known to be involved in β-cell restoration processes—(i) α-cell to β-cell transdifferentiation (9), (ii) neogenesis from multipotent ductal progenitors (1), and (iii) self-replication of pre-existing β-cells (10)—and resulted in the identification of liraglutide as a repurposed drug. As mentioned, liraglutide is an aGLP-1 used to treat type 2 diabetes (11, 12). A recent clinical trial demonstrates that liraglutide reduced HbA1c and insulin requirements in patients with long-standing type 1 diabetes (38). Moreover, it has been described that liraglutide improves β-cell function in alloxan-induced diabetic mice (39).

Our data show that liraglutide improves hyperglycemia, even reaching normoglycemia, in NSG mice. This model was selected for its lack of an adaptive immune system, resulting in immunodeficiency, and absence of autoimmunity. This fact allows us to determine the effect of the drug without autoimmunity interferences and reduced heterogeneity. The amelioration of hyperglycemia was also observed upon glucose stimuli (IPGTT) in diabetic mice treated with liraglutide, whereas diabetic non-treated mice remained hyperglycemic. By contrast, the administration of liraglutide to normoglycemic NOD mice resulted in a weak and transient increase of glycemia levels, as opposed to the effects of insulin administration. Taken together, these results indicate that liraglutide ameliorates hyperglycemia both in fasting and fed conditions but acting differently to insulin. The acute effect of liraglutide on a different mouse strain was insulinotropic as expected. These differences between NOD and C3 mice could be due, at least in part, to genetic differences in the structure and the size of the endocrine pancreas in both strains, specifically in the α- and β-cell mass (40). Another influencing factor should be the islet leukocyte infiltration, a feature of the NOD model, which promotes an inflammatory microenvironment, thus affecting insulin metabolism. The acute effect of liraglutide on a different mouse strain—C3 mice were normoglycemic and free of insulitis—was insulinotropic as expected. The NOD mice are also normoglycemic, but they display islet leukocyte infiltration despite no signs of overt diabetes having been observed at 8 weeks of age. The observation of spontaneous insulitis is restricted to mice with a diabetogenic genetic background, specifically NOD and NOR strains, and CD-1 outbred mice (31). The C3 mice display non-diabetogenic genetic background, do not develop spontaneously autoimmune diabetes, and may have diabetes that can be induced by STZ treatment (41). In NOD mice, insulitis promotes an inflammatory microenvironment, thus causing β-cell stress and metabolic abnormalities (42, 43). Liraglutide administered to NOD mice may also impair insulin secretion at this stage because of the inflammatory environment in this strain.

Intriguingly, the effect was transient as previously described (30). This effect correlated with the transitory increase in the β-cell mass, confirming that insulin secretion induced by liraglutide (11) is not the only event that contributes to the restoration of normoglycemia. This increase is not due to β-cell hyperplasia but by an increase in the number of insulin^+^ cells and the formation of neo-islets emerging from ducts. Thus, it is reasonable to speculate that the continuous presence of liraglutide is required for the maintenance of islet β-cell mass by promoting the main mechanism responsible for the improvement of blood glucose levels in treated mice, at least during the first 30 days of treatment stages. Elucidating the mechanisms of action of liraglutide in diabetic mice with diabetogenic background will contribute to the design of β-cell regenerative strategies.

To further explore the regeneration mechanism, we then searched for processes described in any of the pathways that liraglutide was predicted to act on. Suggesting transdifferentiation, transient bihormonal cells were found within the islets, a feature of α-cell to β-cell conversion detected after β-cell loss (44). Because glycemia normalization occurs almost immediately after the first injections, newly formed insulin^+^ cells with an α-cell origin could contribute to glucose homeostasis. Our results fit well with the recent demonstration that exendin-4, another aGLP-1, also induces bihormonal cells as a consequence of transdifferentiation (16). Other drugs that induce this type of transdifferentiation also cause extreme islet hyperplasia during the early stages of administration (3), but this fact has not been observed with liraglutide, thus suggesting that liraglutide might act through different pathways. Bihormonal cells detected at 48 h after administration agreed with an increase in both insulin and glucagon transcripts in islets treated *in vitro* with liraglutide. Although hormone colocalization *per se* is not a demonstration of a real conversion from α-cell to β-cell, it is logical to speculate that these cells correspond to emergent β-cells. In mice, α-cells can become insulin-expressing cells after β-cell ablation, thus promoting diabetes recovery. Recent results confirm that human α-cells are able to secrete insulin and reverse diabetes, but surprisingly, these cells express α-cell markers (45). Nevertheless, to prove that liraglutide induces a real conversion from glucagon-producing cells into insulin-producing β-cells—as reported with GLP-1 peptide (16), it would be of interest to perform lineage tracing experiments with transgenic models.

Furthermore, during the treatment and even after removal of liraglutide, insulin^+^ bodies were found to be emerging from CK19^+^ ductal cells that expressed insulin. Whether these neo-islets emerged from specific multipotent progenitors remains to be explored, but it has been proposed that early transdifferentiation promotes later neogenesis from ducts (3, 46). Further experiments are required in order to confirm this, given that the cell population identified in the ductal part are Neurogenin3^+^ progenitors that underwent epithelial-to-mesenchymal transition to differentiate into β-like cells (9, 47). Neo-islets have also been reported in human pancreas (48), including those from patients with type 1 diabetes (49), but this is the first demonstration of insulin^+^ cells with a ductal origin caused by liraglutide. Pdx1, a mature β-cell and endocrine progenitor marker, also depicted a ductal population. Because GLP-1 receptor is also expressed in ductal cells (50), insulin expression could be induced by liraglutide in these cells. These results suggest that the role of this drug in ameliorating hyperglycemia follows a mechanism that is non-exclusive to β-cells and has other players involved, such as islet insulin^+^glucagon^+^ cells and ductal insulin-expressing cells. The herein reported findings go beyond the previously reported beneficial effect of liraglutide in β-cell function, both *in vivo* (39) and *in vitro* in terms of insulin secretion (51). However, neogenesis from ducts was not reported in a previous study due to differences in dosage, disease induction, and especially lineage tracing issues (39). It is difficult to assess which insulin-producing cell types are quantitatively contributing to the improvement of hyperglycemia. First, despite the fact that the percentage of bihormonal cells at 48 h is almost 11% of total insulin^+^ cells, these data reflect a specific stage, and it is still unknown how many insulin^+^ cells are arising from transdifferentiation throughout the treatment. Indeed it is reasonable to speculate that ductal insulin^+^ cells are participating in insulin secretion, but it remains to be investigated if they are glucose responsive. Finally, both a decrease in the percentage of budding islets and the fact that liraglutide is an insulinotropic agent may explain the transient effect in the glycemia after withdrawal.

**Figure 7:**
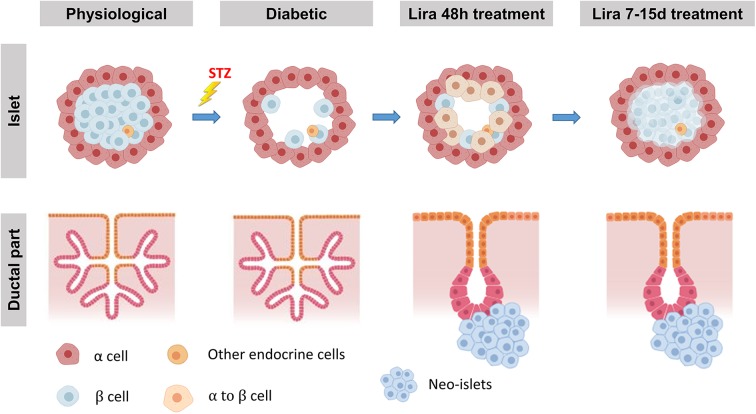
Proposed model of Lira-induced β-cell regeneration. The islets displaying their physiological conditions are depleted from most β-cells when treated with streptozotocin, while the ductal part of the pancreas remains unaffected. We propose that liraglutide can induce a regeneration process in two steps. The first one, triggered at least 48 h after the treatment, consists in the transdifferentiation from mature α-cells into new insulin-expressing β-like cells. During the second step, while β-cell mass seems to be partially recovered, neo-islets formed by insulin-expressing β-like cells from CK19^+^ cells in the ductal part of the pancreas arise from the ducts.

Taking these observations together, we propose a two-step simultaneous mechanism of β-cell regeneration (Figure 7): first, an early, acute, and transient transdifferentiation mechanism from α-cell to β-cell (3, 38) and, second, an early and permanent neogenic process of insulin^+^ cells from the ducts. After withdrawal, the first mechanism seems to be suppressed—probably after the loss of insulin^+^ cell identity—while the second one persists.

In conclusion, liraglutide, a repurposed compound, ameliorates hyperglycemia in experimental type 1 diabetes. Our results point to β-cell replacement, including transdifferentiation and neogenesis, as aiding factor. Liraglutide could be a candidate to restore β-cell mass in combined therapies, together with an immunomodulatory strategy to arrest autoimmunity.

## Data Availability Statement

All datasets generated for this study are included in the article/Supplementary Material.

## Ethics Statement

This study was carried out in strict accordance with the recommendations in the Guide for the Care and Use of Laboratory Animals of the Generalitat de Catalunya, Catalan Government. The protocol was approved by the Committee on the Ethics of Animal Experiments of the Germans Trias i Pujol Research Institute (Permit DAAM 9521) and has followed the principles outlined in the Declaration of Helsinki for animal experimental investigation.

## Author Contributions

AV, FV, IP-A, and MV-P designed the experiments. MC conducted the drug reposition analysis. AV, DP-B, SR-F, and R-MA performed the experiments in mice. AV, DP-B, SR-F, and LG-M carried out the *in vitro* experiments. MC-S bound the drug to the fluorochrome. JV contributed to islet isolation. AV and MV-P wrote the manuscript. SR-F, IP-A, EA, and JV contributed to the discussion. All the authors revised the manuscript and gave final approval of the version to be published.

## Conflict of Interest

MC-S and MV-P are co-founders of Ahead Therapeutics SL, which aims at the clinical translation of immunotherapies for the treatment of autoimmune diseases. MC was employed by the company Anaxomics Biotech SL. The remaining authors declare that the research was conducted in the absence of any commercial or financial relationships that could be construed as a potential conflict of interest.

## Abbreviations

ANN: artificial neural network
AUC: area under the curve
aGLP-1: analog glucagon-like peptide-1
NSG: immunodeficient NOD.SCID-IL2Rγ^−/−^ mice
IPGTT: intraperitoneal glucose tolerance test
ITT: insulin tolerance test
STZ: streptozotocin.
