# Supplementary material for: Repurposed Analog of GLP-1 Ameliorates Hyperglycemia in Type 1 Diabetic Mice Through Pancreatic Cell Reprogramming
Source: Front Endocrinol (Lausanne). 2020 May 13;11:258. doi: 10.3389/fendo.2020.00258 (PMC7237704; doi:10.3389/fendo.2020.00258)
Supplement: Supplementary file 1 [file Data_Sheet_1.docx]

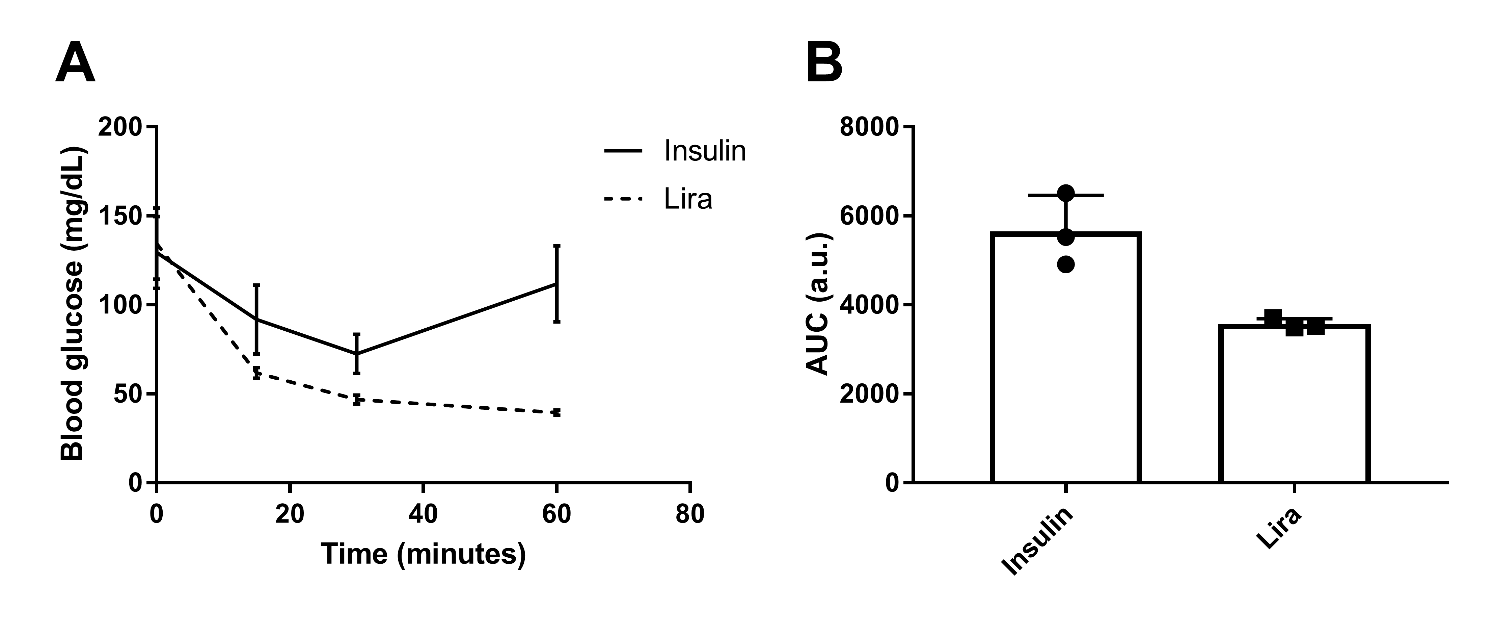


**Supplementary Figure 1. Effect of liraglutide in normoglycaemic C3HeB/FeJ mice. A)** Insulin Tolerance test (ITT) performed in normoglycaemic mice injected with Lira (1mg/kg, dashed line) or insulin (0.5 U/kg, continuous line). **B)** AUC of the graph in A). Results are mean ± SD, no statistical differences were found between groups.

**
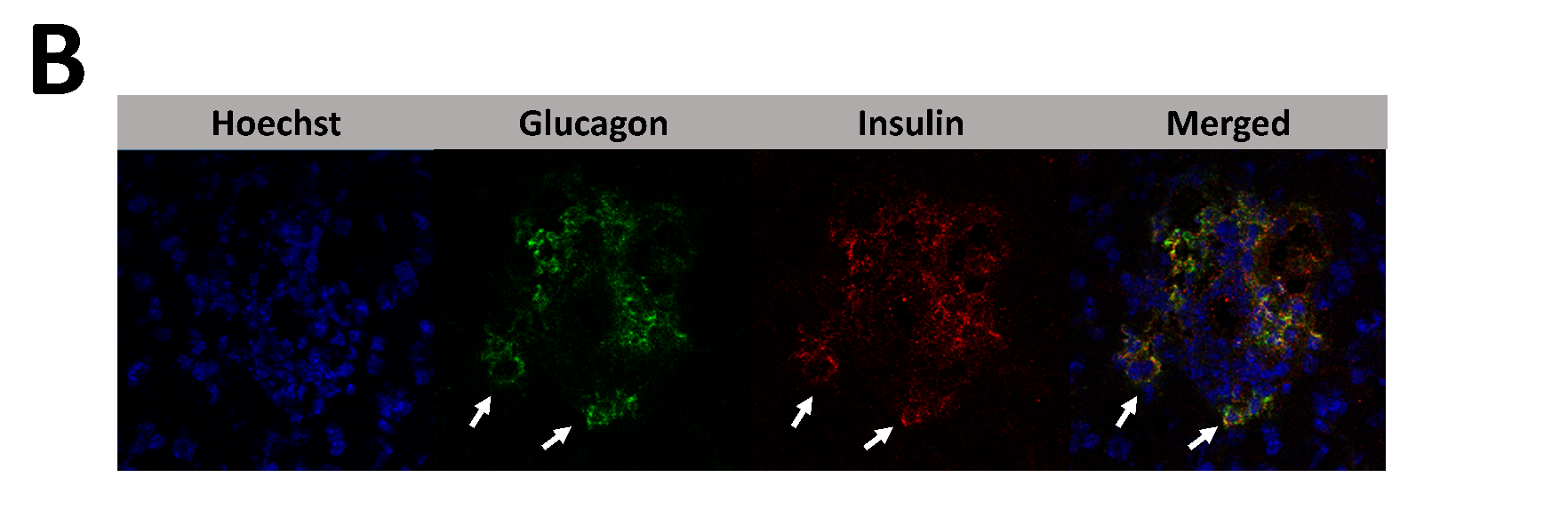
**

**Supplementary Figure 2. Colocalization of insulin and glucagon in bihormonal insulin^+^glucagon^+^ cells.** Colocalization of glucagon and insulin in a pancreatic islet of a Lira 48h mouse revealed insulin^+^glucagon^+^ cells (white arrows). Section of 1µm thickness was recorded at 40X with a confocal microscope.

**
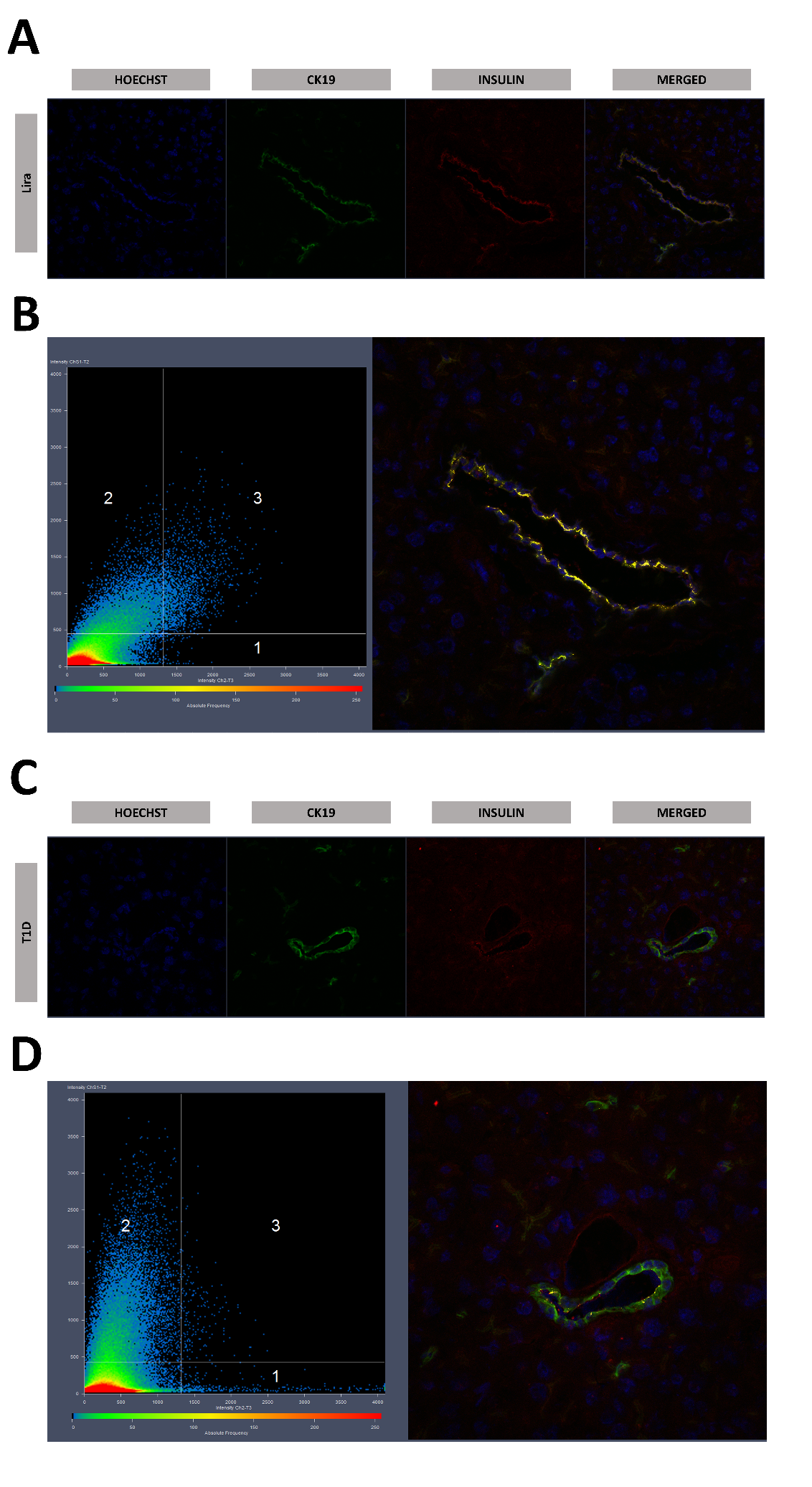
**

**Supplementary Figure 3. Colocalization of insulin and CK19 in CK19^+^insulin^+^ ductal cells. A)** Colocalization of CK19 (green) and insulin (red) in a ductal area from a pancreas of a Lira mouse revealed insulin^+^ ductal cells. **B).** Colocalization dot plot of CK19 (y-axes) and insulin (x-axes) of the picture in A). Colocalization area highlighted in yellow pseudocolor (Zen Black, Zeiss). **C).** No colocalization of CK19 (green) and insulin (red) was observed in ductal cells from pancreases from non-treated T1D mice. **D).** Colocalization dot plot of CK19 (y-axes) and insulin (x-axes) of the picture in C) (Zen Black, Zeiss). Sections of 1µm thickness were recorded at 40X with a confocal microscope (Axiobserver Z1, Zeiss).
