# Supplementary material for: Repurposed Analog of GLP-1 Ameliorates Hyperglycemia in Type 1 Diabetic Mice Through Pancreatic Cell Reprogramming
Source: Front Endocrinol (Lausanne). 2020 May 13;11:258. doi: 10.3389/fendo.2020.00258 (PMC7237704; doi:10.3389/fendo.2020.00258)
Supplement: Supplementary file 2 [file Table_1.docx]

**Supplementary table 1. Effector proteins selected for the *Drug Repositioning* analysis.**

| Pathway | Effector Protein | Uniprot ID |
| --- | --- | --- |
| Self-replication | INGAP | Q92778 |
| Self-replication | PTF1A | Q7RTS3 |
| Self-replication | MYC | P01106 |
| Self-replication | CPA1 | P16085 |
| Self-replication | NEUROG3 | Q9Y4Z2 |
| Self-replication | MKI67 | P46013 |
| Self-replication | CCND2 | P30279 |
| Self-replication | CDK4 | P11802 |
| Self-replication | E2F1 | Q01094 |
| Self-replication | AKT1 | P31749 |
| Self-replication | KMT2A | Q03164 |
| Self-replication | SLC2A2 | P11168 |
| Self-replication | INS | P01308 |
| Self-replication | PDX1 | P52945 |
| Self-replication | MAFA | Q8NHW3 |
| Self-replication | NEUROD1 | Q13562 |
| Self-replication | GCK | P35557 |
| Self-replication | ISL1 | P61371 |
| Self-replication | NOTCH1 | P46531 |
| Self-replication | REG1B | P48304 |
| Self-replication | REG1A | P05451 |
| Neogenesis | PDX1 | P52945 |
| Neogenesis | SOX9 | P48436 |
| Neogenesis | SLC2A2 | P11168 |
| Neogenesis | NEUROG3 | Q9Y4Z2 |
| Neogenesis | SST | P61278 |
| Neogenesis | MSX2 | P35548 |
| Neogenesis | EGF | P01133 |
| Neogenesis | FBXW7 | Q969H0 |
| Neogenesis | STAT3 | P40763 |
| Neogenesis | IL6 | P05231 |
| Transdifferentiation | PAX4 | O43316 |
| Transdifferentiation | GCG | P01275 |
| Transdifferentiation | NEUROG3 | Q9Y4Z2 |
| Transdifferentiation | GLP1R | P43220 |
| Transdifferentiation | ARX | Q96QS3 |
| Transdifferentiation | PDX1 | P52945 |
| Transdifferentiation | NKX2-5 | P52952 |
| Transdifferentiation | FOXO1 | Q12778 |
| Transdifferentiation | CDK4 | P11802 |
| Transdifferentiation | PAX6 | P26367 |
| Transdifferentiation | NKX6-1 | P78426 |

In order to perform the repositioning of approved drugs, selected list of proteins known to be involved in the following pathways —(i) self-replication from pre-existing β cells, (ii) neogenesis from ductal progenitors and (iii) transdifferentiation from α-cells to β-cells— was obtained.
