# Supplementary material for: Repurposed Analog of GLP-1 Ameliorates Hyperglycemia in Type 1 Diabetic Mice Through Pancreatic Cell Reprogramming
Source: Front Endocrinol (Lausanne). 2020 May 13;11:258. doi: 10.3389/fendo.2020.00258 (PMC7237704; doi:10.3389/fendo.2020.00258)
Supplement: Supplementary file 3 [file Table_2.DOCX]

**Supplementary table 2. Histometric data from NGS mice treated or not with Lira.**

| Parameter | STZ | Lira ongoing | Lira ended | Sham |
| --- | --- | --- | --- | --- |
| Body weight (g) | 27.95±0.41 | 25.68±4.53 | 26.47±2.41 | 26.93±0.80* |
| Pancreas weight (mg) | 122±15.83 | 128.8±37.41 | 141.2±14.48 | 114±20.21* |
| Pancreas weight/ g of body weight | 4.36±0.57 | 5.05±1.21 | 5.38±0.91* | 4.01±0.71 |
| β cell % of pancreatic tissue | 1.45±0.50 | 2.73±0.87* | 1.30±0.65 | 4.94±1.44*** |
| β cell number/mg of pancreatic tissue | 975.3±294.5 | 1355±911.4 | 596±167.9* | 2266±691.9** |
| β cell number/g of body weight | 4254±1411 | 6683±4587 | 3106±687.3 | 9280±2197*** |
| Insulin+ aggregates | 2686±798.8 | 5138±1842* | 2272±932.6 | 4991±1207** |

Histometry from NSG mice rendered diabetic by a single-dose STZ treated with PBS (STZ), daily s.c. Liraglutide for 7-15 days (Lira ongoing), after the withdrawal (Lira ended) and a normoglycemic group (Sham). Data presented as mean ± SD. Asterisks denote statistical significance when comparing each parameter in any group with the STZ group (*p < 0.05, **p < 0.01, ***p < 0.001, ****p < 0.0001, Mann-Whitney test).
